# Bioinformatic Analysis, Molecular Modeling of Role of Lys65 Residue in Catalytic Triad of D-aminopeptidase from *Ochrobactrum anthropi*

**Published:** 2010-07

**Authors:** I.G. Khaliullin, D.A. Suplatov, D.N. Shalaeva, M. Otsuka, Y. Asano, V.K. Švedas

**Affiliations:** Faculty of Bioengineering and Bioinformatics, Lomonosov Moscow State University; Department of Chemistry, Lomonosov Moscow State University; Biotechnology Research Center, Toyama Prefectural University, Japan

**Keywords:** D-aminopeptidase, penicillin-binding protein family, bioinformatic analysis, catalytic mechanism

## Abstract

A bioinformatic and phylogenetic study has been performed on a family of penicillin–binding proteins including D–aminopeptidases, D–amino acid amidases, DD–carboxypeptidases, and β –lactamases. Significant homology between D–aminopeptidase from *Ochrobactrum anthropi* and other members of the family has been shown and a number of conserved residues identified as S62, K65, Y153, N155, H287, and G289. Three of those (Ser62, Lys65, and Tyr153) form a catalytic triangle – the proton relay system that activates the generalized nucleophile in the course of catalysis. Molecular modeling has indicated the conserved residue Lys65 to have an unusually low pKa value, which has been confirmed experimentally by a study of the pH–profile of D–aminopeptidase catalytic activity. The resulting data have been used to elucidate the role of Lys65 in the catalytic mechanism of D–aminopeptidase as a general base for proton transfer from catalytic Ser62 to Tyr153, and *vice versa*, during the formation and hydrolysis of the acyl – enzyme intermediate.

## INTRODUCTION

D–aminopeptidase from *Ochrobactrum anthropi* possesses a unique structural organization, high stereospecificity, and shows catalytic activity towards a wide range of D–alanine derivatives [1]. It is a member of the serine hydrolases superfamily and acts as a homodimer, with each subunit consisting of three structural domains. One of those – a catalytic domai n – has the so called β –lactamase fold [2]. Sequence comparison has revealed a strong evolutionary relationship of D–aminopeptidase with DD–carboxypeptidase and β –lactamase [3].

The catalytic mechanism of D–aminopeptidase has not been discussed in the literature; however, suggestions concerning other enzymes of the family – D–amino acid amidase from *Ochrobactrum anthropi*, as well as β–lactamases and penicillin–binding proteins, can be taken into account. Different views on the catalytic mechanism of penicillin–binding proteins are presented, and several residues in close proximity to the catalytic serine are considered as potential candidates for the role of general base in the course of the enzymatic reaction [4– 6]. While choosing between them, it is important to look at the pH–profile of enzyme activity and possible pK shifts of the residue due to its environment, since the general base is bound to act in deprotonated form.

It is important that molecular modeling of the deacylation step has been performed for reactions catalyzed by DD–peptidase R61 from * Streptomyces sp. * and class С β –lactamase P99 from * Enterobacter cloacae * by means of QM/MM methods [7]. It revealed the leading role of the active site tyrosine residue in the deacylation of the catalytic serine of those enzymes.

Yet, an analysis of the literature shows that a comprehensive view on the catalytic mechanism of D–aminopeptidase is still lacking. In this work, we aim to use bioinformatics and molecular modeling to elucidate the role of the Lys65 residue in the catalytic triad of D–aminopeptidase from *Ochrobactrum anthropi*.

## MATERIALS AND METHODS

**Experimental study**. D–aminopeptidase was purified according to a procedure described earlier [1]. The colorimetric substrate – D–alanine * p * –nitroanilide (D–Ala– *p*NA) – was produced by Bachem. Tris(hydroxymethyl)methylamine (Tris) of research–grade purity produced by SERVA Electrophoresis was used to prepare buffer solutions.

Kinetic assays were performed using progress curve analysis. The studies were carried out in a spectrophotometric cuvette of 500 μl and optical path length of 1 cm thermostated at 25°C. Enzymatic hydrolysis was initiated by adding a small amount of the enzyme solution to the reaction mixture containing the substrate in a concentration approximately 4 times higher than its K_M_. Changes in absorption were registered by a Shimadzu UV–1601 spectrophotometer in the Kinetics mode at 450 nm. The final levels of absorption were not higher than 2 in all of the assays.

To maintain the set pH value in the reaction mixture, the enzymatic reactions were carried out in a 0.1 M Tris–HCl buffer. A Hamilton Slimtrode pH–sensitive electrode was used in the preparation of the buffer solutions.

The progress curves obtained were processed by data linearization in *t/ln(p_∞_/(p_∞_–p)) – p/ln(p_∞_/(p_∞_–p)) * coordinates, where * t* is time, * p * – current concentration of the product, and *p_∞_* – the final concentration of the product. This anamorphosis allows to determine the value of the K_M_/V_max_ and 1/V_max_ ratio as the line’s slope and * y * –intercept, respectively. The nonlinear regression of the V_max_/K_M_ dependence on pH and the computation of experimental pKa values were performed using SciDAVis software [8].

**Homology search**. In all homology search procedures, a sequence or structure of D–aminopeptidase from *Ochrobactrum anthropi* (PDB entry 1EI5) was used as a query.

The sequence–based homology search was carried out using the PSI–BLAST [9] algorithm v. 2.2.18 to scan a “non–redundant” protein sequence database. The resulting sample was filtered with a 95% pairwise identity threshold to eliminate redundancy and then aligned using t_coffee [10], mafft [11], and probcons [12] joined together by the consistency–based statistics implemented in t_coffee.

The structure–based homology search was carried out by scanning the PDB protein structure databank using the SSM [13] procedure. Hits were discriminated in case of high secondary structure elements mismatch with the 1EI5 structure. The resulting sample of three–dimensional structures was aligned using the MUSTANG [14] software.

Bioinformatic analysis. The phylogenetic analysis of sequence and structural alignment was performed using the phylip package [15]. The phylograms were constructed using distance–based methods with the neighbor–joining algorithm. The bioinformatic analysis was carried out using the original ZEBRA v. 3.2 software with a statistical threshold level of 2.2 × 10^–43^.

Visualisation. The Jalview [16] program was used to look through multiple sequence alignments. Visualization of three–dimensional structures and a structure–based multiple alignment was done using PyMol [17]. Generation of phylogenetic trees was done using phylip [15]. Generation of sequence patterns logotypes was done with the WebLogo [18] Internet service.

## RESULTS AND DISCUSSION

**Fig. 1:**
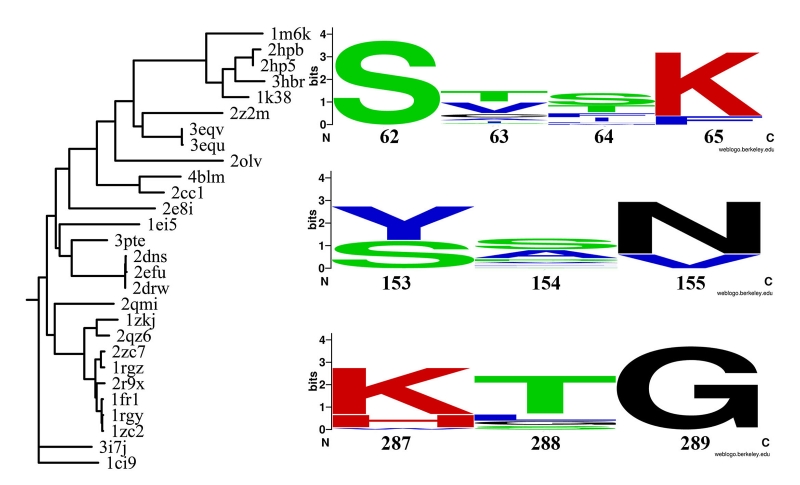
Phylogenetic tree based on structural alignment of penicillin-binding proteins and primary structure motives containing important catalytic residues: Ser62 and Lys65 (SXXK), Tyr153 ([YS]XN) and His287 ([KH] XG). Leaves are named according to PDB databank accession numbers.

**Bioinformatic analysis of enzymes homologous to D–aminopeptidase**. Data related to the penicillin–binding protein family including D–aminopeptidases, D–amino acid amidases, and alkaline D–peptidases were collected and analyzed. The UniProt protein sequence database and PDB protein structures databank were screened * a priori * to identify all significant sequences and structure–based homologs of D–aminopeptidase. The resulting set of 734 sequence homologs and 24 structural homologs was sampled and filtered to acquire the most informative set. As a result of structural alignment, a significant similarity of the active site regions of D–aminopeptidase, alkaline D–peptidases, D–amino acid amidases, and β –lactamases was shown for both the sequence and structure levels (Fig. 1).

**Fig. 2:**
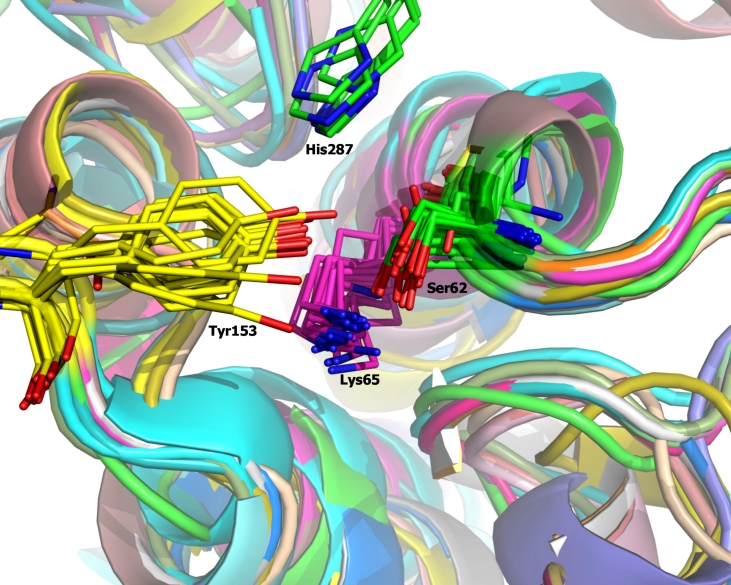
An active site fragment of the structural alignment of penicillin-binding proteins – structural homologs of D-aminopeptidase from Ochrobactrum anthropi. Location of four conserved active site residues — Ser62, Lys65, Tyr153, His287-conserved in D-aminopeptidase, as well as in other members of the family, is shown.

The following residues were identified as conserved in the active site of penicillin–binding proteins using the original ZEBRA software developed in our laboratory: 287H, 153Y, 155N, 289G, 273G, 293G, 270Y, 65K, 224G, 62S, 68T, 294W, 64S, 151Y, 228I, 60I, and 288G (numbered according to the 1EI5 structure and sorted in decreasing significance). Considering that the conservation of a residue in a protein structure indicates an evolutionary pressure on that position and thus underlines its functional or structural importance [19], we suggest that such residues are important to the D–aminopeptidase catalytic mechanism. A common alpha–beta domain identified as a “three–layer sandwich” by CATH [20] structure classification was shown to contain active site residues in all studied penicillin–binding proteins (Fig. 2).

**Fig. 3:**
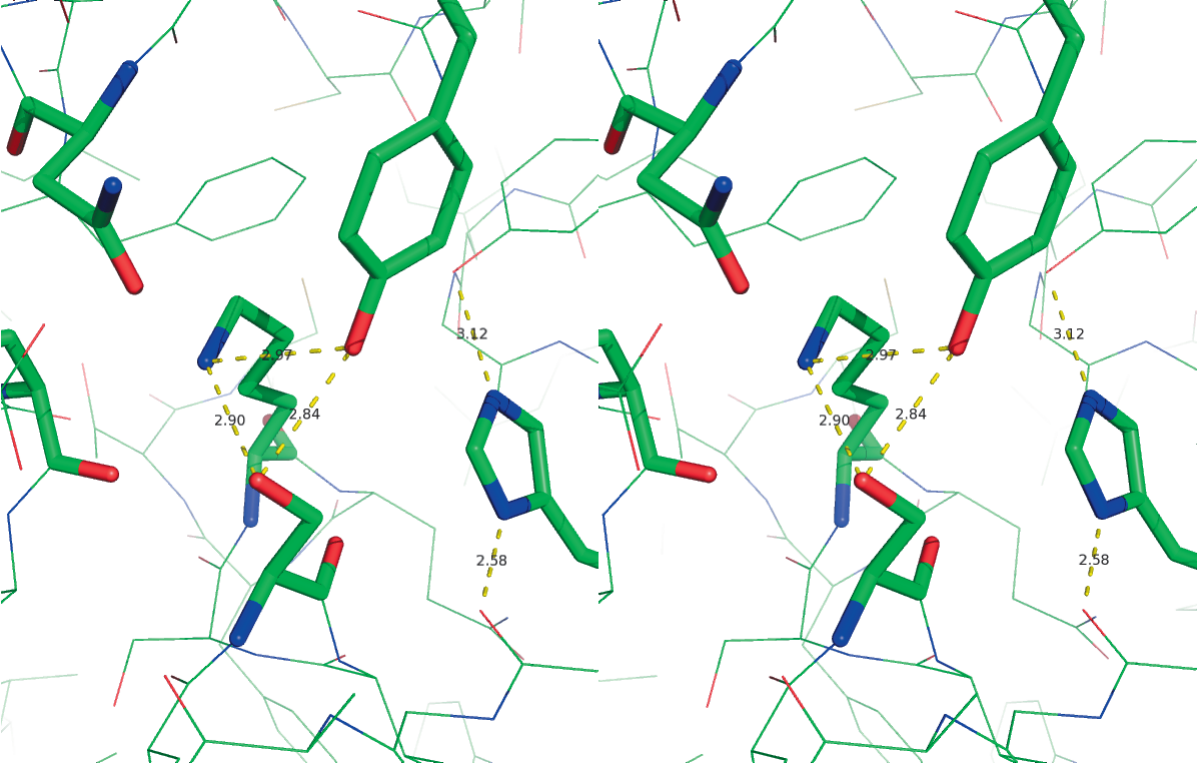
Stereo view of the D-aminopeptidase active site and spatial organization of catalytic triad. The S62, K65, Y153, N155, H287, and D225 residues are highlighted.

**D-aminopeptidase structure analysis**. The D–aminopeptidase structure (PDB entry 1EI5) analysis reveals a pair of amino acid residues, Tyr153 and Lys65, located in near proximity and in approximately equal distance to the catalytic Ser62’s O γ atom. All three residues form a nearly equilateral triangle in the enzyme active site (Fig. 3). Such a location of residues implements a special organization of the proton relay system when the hydrogen atom of the serine’s hydroxyl–group is directed toward the center of the triangle and shared among all of the residues of the catalytic triad. The specific organization of this catalytic triangle is based on the irregular properties of the Lys65 residue capable of accepting a proton in neutral and slightly alkaline media, which lends high reactivity to the Ser62 residue at the formation of the acyl – enzyme intermediate.

A considerably lower pKa value of the Lys65 residue equal to 7.8, compared to the ionization of regular lysine residues in proteins with pKa 10–11, was observed upon calculation of the ionization properties of D–aminopeptidase active site residues by the PROPKA QSAR method [21]. A high–evaluated pKa value of the Tyr153 residue equal to 11.85 should be noted as well.

**Experimental pH-profile of D-aminopeptidase catalytic activity**

**Fig. 4:**
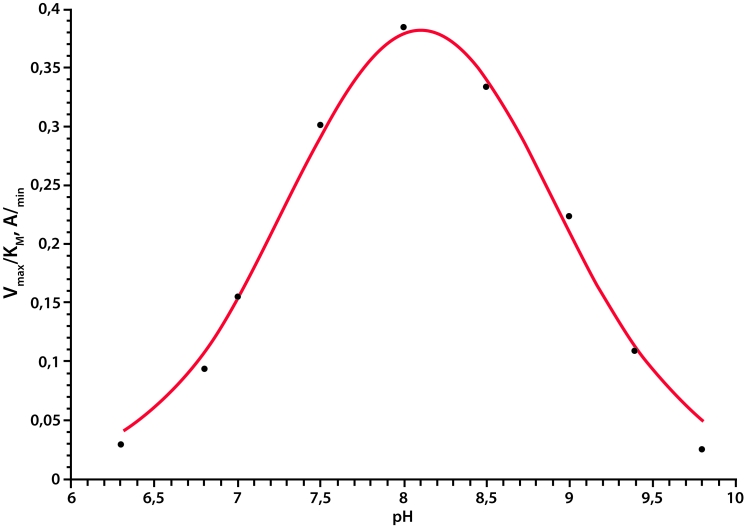
pH-profile of D-aminopeptidase catalytic activity. Theoretical curve was calculated according to the equation v = v_0_/ (1+[H^+^]/K_1_+K_2_/[H+]), where v_0_, pK_1_ and pK_2_ values are equal to 0.53, 7.4 and 8.8, respectively.

Experimental data are in good agreement with the results obtained by molecular modeling and shed light on the role of the Lys65 residue in the functioning of the catalytic triad. The pH–profile of the D–aminopeptidase catalytic activity has a bell–shaped form with a pKa value of 7.4 and a pKb value of 8.8 (Fig. 4). On the basis of the molecular modeling and the experimental study, the amino acid residue with a pKa of 7.4 was referred to Lys65.

**Fig. 5:**
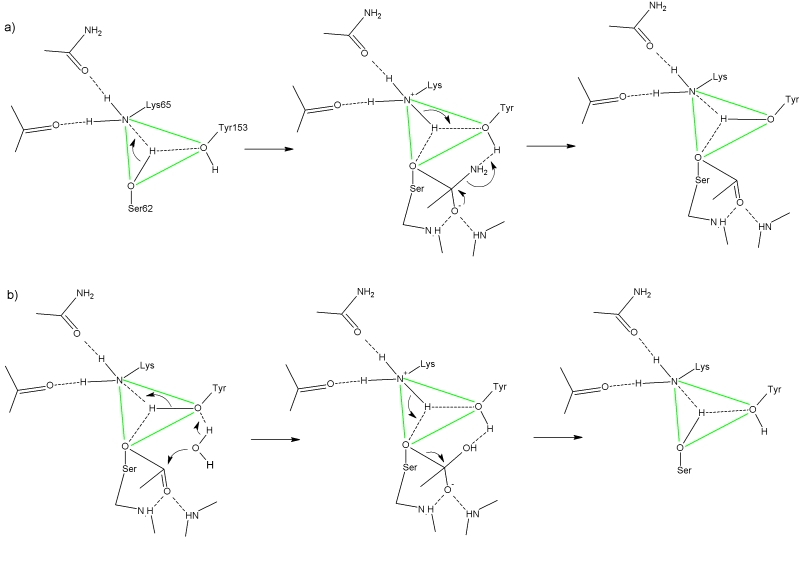
Schematic presentation of acylation (a) and deacylation (b) of the Ser62 residue in catalytic mechanism of D-aminopeptidase: a) organization of catalytic triad and its role in formation of the first tetrahedral intermediate followed by formation of acyl-enzyme are shown; b) nucleophile (water molecule) binding, activation, nucleophilic attack followed by formation of the second tetrahedral intermediate and regeneration of free enzyme are shown.

**Proposed catalytic mechanism of D–aminopeptidase**. Just as in the case of other serine hydrolases, the reaction catalyzed by D–aminopeptidase follows a 3–step kinetic scheme with the formation of a covalent acyl–enzyme intermediate and its subsequent hydrolysis or transfer of the acyl group to an external nucleophile. Because of the extremely low pKa value of its terminal amino group, the Lys65 residue plays a specific role in the D–aminopeptidase catalytic mechanism (Fig. 5):

Being uncharged at the pH–optimum of the enzymatic reaction, the Lys65 residue acts as a general base, while the O γ atom of Ser62 attacks the carbonyl group of a substrate: Lys65 assumes a proton from the attacking OH–group when the first tetrahedral intermediate is formed at the acylation step.

At the decomposition of the first tetrahedral intermediate followed by the formation of the acyl–enzyme and release of the first reaction product, its leaving group gathers a proton donated by the OH–group of Tyr153, whose acidity is increased due to the proximity of a positively charged Lys65 residue, and at the same time the formed oxyanion of Tyr153, being stronger base, captures a proton from the Lys65 residue.

At the deacylation step, the water molecule (or molecule of another nucleophile) is activated through the concerted action of two bases – Tyr153 and Lys65. Protons are transferred by the proton relay system from the nucleophile to Tyr153 and from Tyr153 to Lys65, and a nucleophilic attack occurs, followed by the formation of the second tetrahedral intermediate.

At the decomposition of the second tetrahedral intermediate followed by the release of the second reaction product, the Lys65 residue cedes a proton to the Ser62 oxyanion and the enzyme returns to its initial state.

## CONCLUSIONS

The bioinformatic and phylogenetic analysis of the penicillin–binding protein family including D–aminopeptidases was carried out, and the conserved residues were identified. Three of them – catalytic Ser62, Lys65, and Tyr153 – form a catalytic triangle – a specific proton relay system that captures/cedes a proton in the course of catalytic events and makes possible the activation of the generalized nucleophile in the D–aminopeptidase catalysis. Molecular modeling showed that a conserved residue (Lys65) possess an unusually low pKa value, which was confirmed by an experimental pH–profile of the D–aminopeptidase catalytic activity. The specific role of the Lys65 residue in a catalytic mechanism of D–aminopeptidase was proposed at the formation and hydrolysis of the acyl – enzyme intermediate.
